# Supplementary material for: Content-rich biological network constructed by mining PubMed abstracts
Source: BMC Bioinformatics. 2004 Oct 8;5:147. doi: 10.1186/1471-2105-5-147 (PMC528731; doi:10.1186/1471-2105-5-147)
Supplement: Additional File 5 — The original Chilibot query results of the term "long-term potentiation (LTP)" and 22 other terms, limiting the latest references analyzed to the years 1990, 1995, 2000, and 2004. [file 1471-2105-5-147-S5.bz2 › chilibotAdditionalFile5/ltp1995/html/PKA_ACTININ.html]

 


 **PKA** and **ACTININ** 
  
Found 1 abstracts in PubMed,  **1 abstracts were retrieved and analyzed**.  


---

 Search Google  |
 PDF files only 
|  EDU domain only 

---

- Cell Motil Cytoskeleton, 1994   **Regulation of motility and cytoskeletal organization of rat bladder carcinoma cells by cyclic AMP.** .
  Cyclic AMP cAMP has been implicated in the regulation of movement of certain cultured cell types.
  We have studied the effects of cAMP on epithelial cell motility using serum free NBT II cells, derived from a rat bladder carcinoma.
  The random movement of these cells on type I collagen was reduced upon elevation of intracellular cAMP by several means and this effect was reversible.
  Alterations in the organization of the cytoskeletal proteins F actin and alpha **actinin** occurred concurrently with the reduction in motility, and the arrangement of these proteins resembled that seen in non motile cells on glass.
  In addition, pretreatment of cells with KT5720, a cAMP dependent protein kinase **PKA** specific inhibitor, prevented the dibutyryl cAMP induced reduction in cell movement as well as the associated cytoskeletal changes.
  These results suggest that elevation of **PKA** is responsible for the observed effects on cell motility and cytoskeletal reorganization and demonstrate a role for **PKA** in the regulation of cell motility in this system.
